# A Rare Case of Ventricular Septal Defect and Double-Chambered Right Ventricle With Bilateral Cardiac Infective Endocarditis

**DOI:** 10.7759/cureus.79749

**Published:** 2025-02-27

**Authors:** Idriss Souko, Tarek Abdel Aziz, Mohannad Alasaad, Zohair Al-Halees, Obaid Aljassim

**Affiliations:** 1 Cardiothoracic Surgery, Dubai Hospital, Dubai, ARE; 2 Cardiac Surgery, King Faisal Specialist Hospital and Research Centre, Riyadh, SAU

**Keywords:** double-chambered right ventricle, infective endocarditis, multivalvular endocarditis, septic pulmonary emboli, vsd

## Abstract

Infective endocarditis (IE) typically affects the left side of the heart. Multiple valve involvement on both sides of the heart is a rare occurrence. However, congenital heart defects, particularly ventricular septal defects (VSD), can predispose individuals to bilateral endocarditis. We present a case of a 41-year-old patient with congenital VSD and double-chambered right ventricle (DCRV) who presented with fever, generalized fatigue, and unintentional weight loss of 20 kg over two months. Transthoracic echocardiography revealed vegetations on the aortic valve, pulmonary valve, and right ventricular outflow tract (RVOT), alongside a small VSD with left-to-right shunt. The blood cultures revealed *Streptococcus mutans*. Initially, the patient was treated with intravenous antibiotics. In the further course, she showed persistent symptoms and signs of sepsis and failure of vegetation regression. The decision was made for surgical intervention. The aortic valve was replaced with a mechanical valve (St. Jude Medical Regent, 23 mm, Saint Paul, Minnesota, United States), and the pulmonary valve was replaced with a tissue valve (Medtronic Freestyle, 27 mm, Minneapolis, Minnesota, United States). The VSD was closed directly, and the fibromuscular tissue causing DCRV was resected. The postoperative recovery was uneventful, and the patient was discharged in stable general condition. This case emphasizes the importance of considering congenital heart defects in patients with bilateral endocarditis, highlighting the need for comprehensive preoperative echocardiographic evaluation and intraoperative assessment. Endocarditis prophylaxis should be strongly considered for patients with intracardiac shunts, particularly during dental procedures involving the disturbance of the gingival tissue, the periapical region of teeth, or the oral mucosa.

## Introduction

Ventricular septal defects (VSDs) are the most prevalent congenital heart disease [1]. They can occur as isolated defects, representing 20% of all VSDs, or in combination with other congenital anomalies [2]. The natural progression of VSDs varies, with some undergoing spontaneous closure, while others persist from infancy into adolescence. Persistent VSDs can lead to complications such as left ventricular overload, increased pulmonary vascular resistance, or aortic valve insufficiency [3].

Double-chambered right ventricle (DCRV) is a condition characterized by the division of the right ventricle into a high-pressure tricuspid side and a low-pressure pulmonary side. This pressure gradient can create a jet force that damages the endocardial lining of the right ventricular outflow tract (RVOT) distal to the stenotic region, thereby increasing the risk of endocarditis. This may subsequently lead to pulmonary septic embolization and infarction. DCRV is associated with VSD in approximately 77% of cases [4].

Congenital VSDs are associated with an increased risk of infective endocarditis (IE) compared to individuals without such defects [5-7]. However, multivalvular IE involving both cardiac chambers is rare, and it is mostly associated with congenital heart defects [8]. 

According to the 2023 European Society of Cardiology (ESC) guidelines, IE can be managed medically in many cases. However, surgical intervention is indicated in the presence of heart failure or uncontrolled infection or to prevent septic embolization [9].

Careful preoperative assessment with echocardiography and intraoperative evaluation for intracardiac shunts are essential for detecting these lesions and ensuring proper treatment. Failure to identify and manage these lesions may increase postoperative morbidity and mortality.

We present a rare case of bilateral cardiac endocarditis with VSD and DCRV.

## Case presentation

A 41-year-old female patient with a history of unknown congenital heart defect since childhood presented with hemoptysis, fatigue, and a 20 kg weight loss over the last two months. On presentation, she was somnolent, febrile (temperature: 39.1°C), and hypotensive (blood pressure: 87/62 mmHg; heart rate: 131 beats/min; respiratory rate: 25/min). Immediate resuscitation was performed. Initial laboratory studies were significant for anemia and elevated infection markers (Table 1).

**Table 1:** Laboratory investigations at hospital admission

Laboratory parameters	Results	Reference value
Hemoglobin	5.5 g/dL	12-15 g/dL
Hematocrit	17.5%	36-46%
Mean corpuscular volume	72.7 fl	77-95 fl
Mean corpuscular hemoglobin	23 pg	27-32 pg
Leukocytes	24,000/mm^3^	3,600-11,000/mm^3^
Platelets	356,000/mm^3^	150,000-410,000/mm^3^
C-reactive protein	125.4 mg/L	<5 mg/L

**Figure 1:**
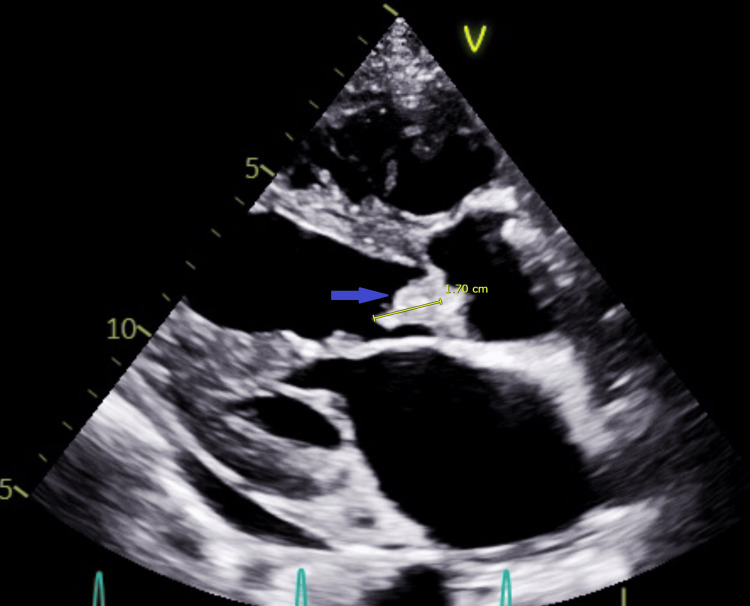
Transthoracic echocardiogram parasternal long-axis view showing a 17 mm vegetation on the aortic valve (blue arrow)

The blood cultures were positive for *Streptococcus mutans*. Transthoracic echocardiography (TTE) showed multiple vegetations on all aortic valve cusps, with the largest measuring 17×11 mm and moderate-to-severe aortic valve regurgitation (Figure 1).

**Figure 2:**
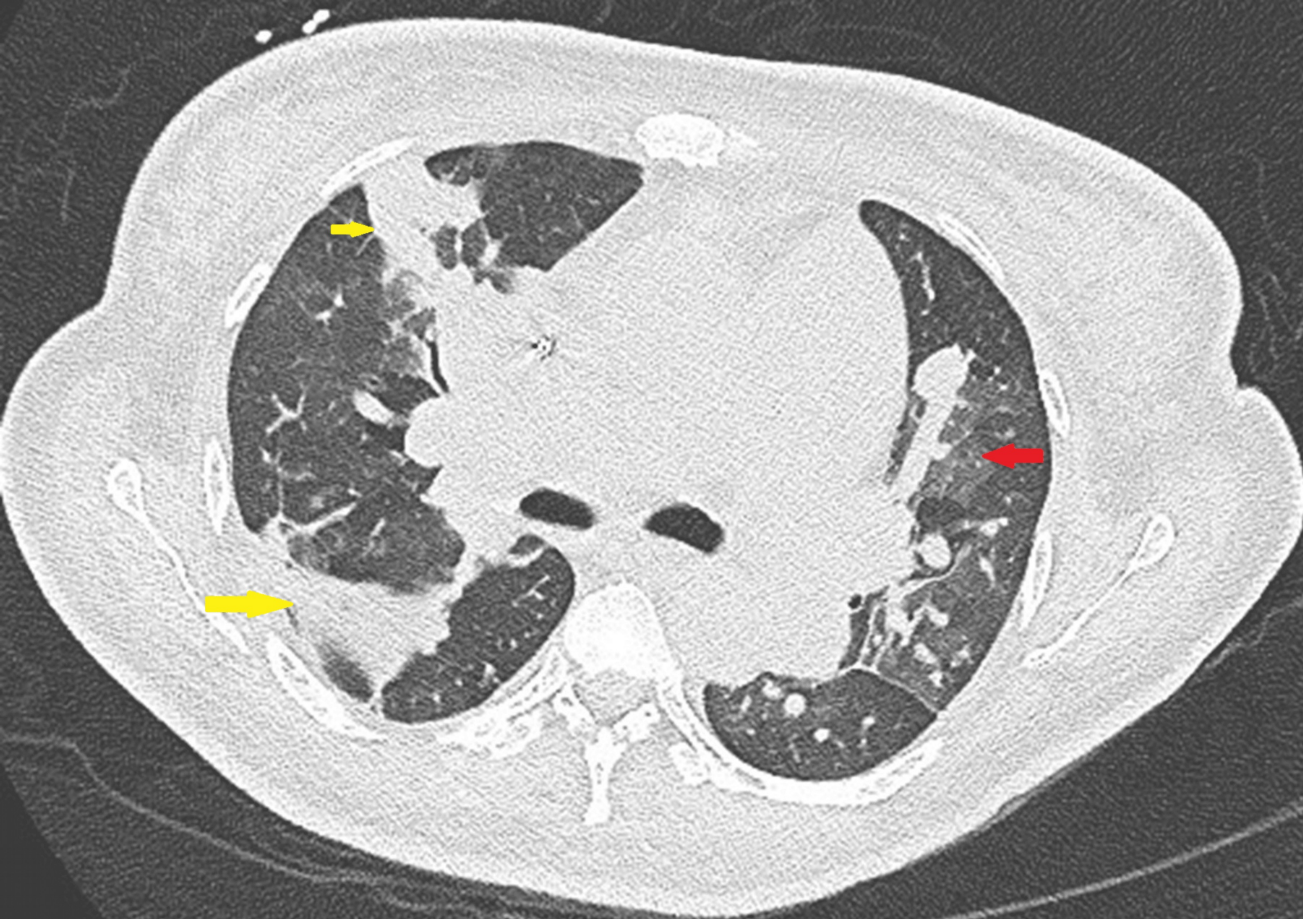
Axial CT scan showing pulmonary septic emboli (yellow arrows) and pulmonary infarction (red arrow) CT: computed tomography

The left ventricular function was mildly reduced. In addition, there were multiple vegetations on the pulmonary valve and in the RVOT. A 6 mm VSD with a left-to-right shunt was also detected. Chest computed tomography (CT) demonstrated evidence of multiple pulmonary emboli with pulmonary infarctions (Figure 2).

The heart team meeting along with consultations from infectious disease specialists recommended conservative treatment with intravenous vancomycin (30 mg/kg/day) and ceftazidime (90 mg/kg/day).

Following blood transfusion, anemia correction, and antibiotic therapy, the patient was initially stabilized. However, over the next two weeks, she gradually developed signs of heart failure, including progressive dyspnea with minimal exertion, generalized edema, and pleural effusions. Follow-up TTE showed no significant changes in the size of the vegetations. After further discussions with cardiology and infectious disease departments, the decision was made to proceed with surgical treatment.

Surgical technique

**Figure 3:**
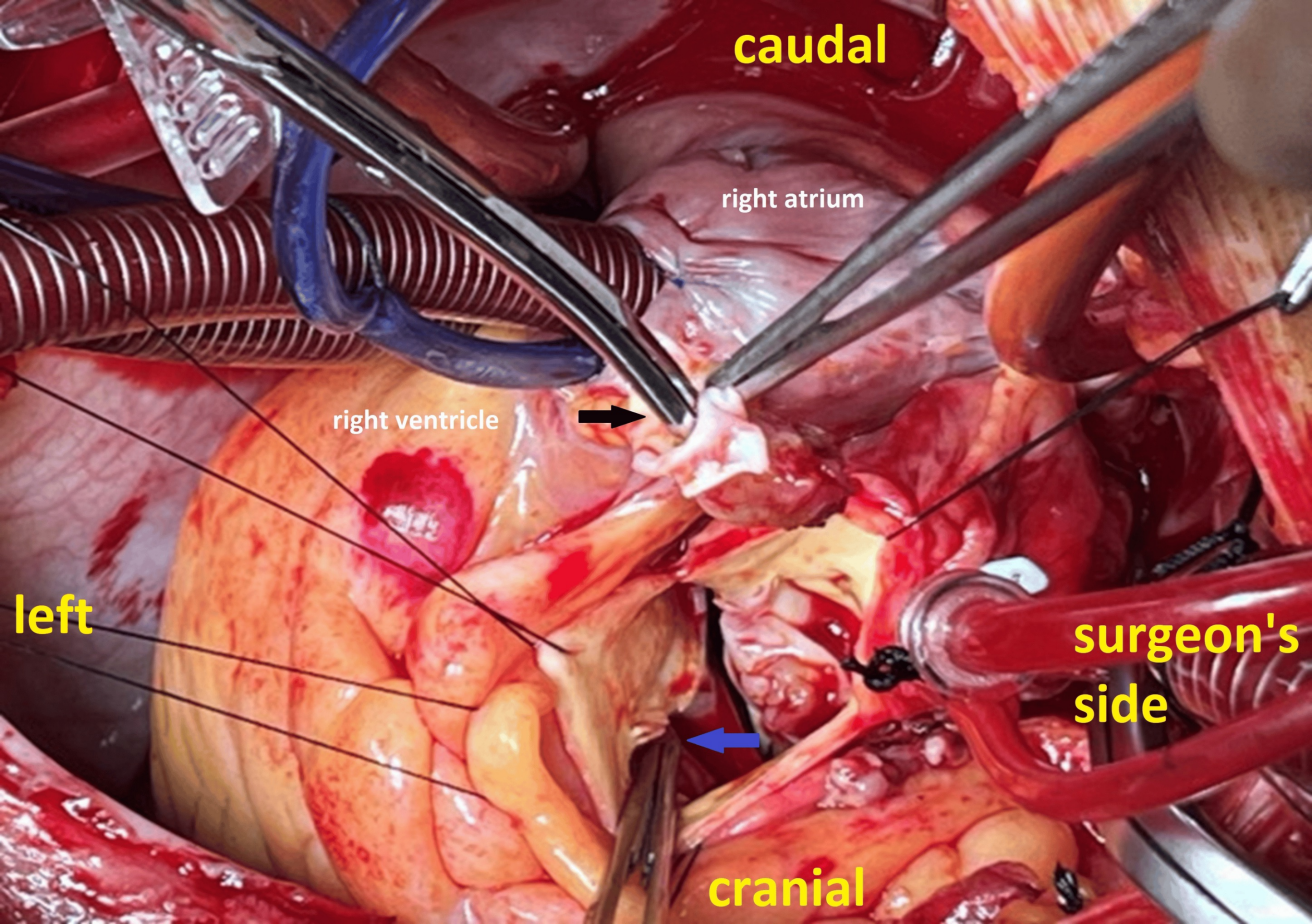
Image showing perforation in the right coronary cusp (the tip of scissors inside the perforation, indicated by a black arrow) and a small VSD (the tip of forceps, blue arrow) VSD: ventricular septal defect

Using a heart-lung machine with aortic and bicaval cannulation, with a cardiopulmonary bypass time of 177 minutes and an aortic cross-clamp time of 110 minutes, multiple vegetations were confirmed on all aortic valve cusps, along with a perforation in the right coronary cusp. A small VSD was also identified (Figure 3).

**Figure 4:**
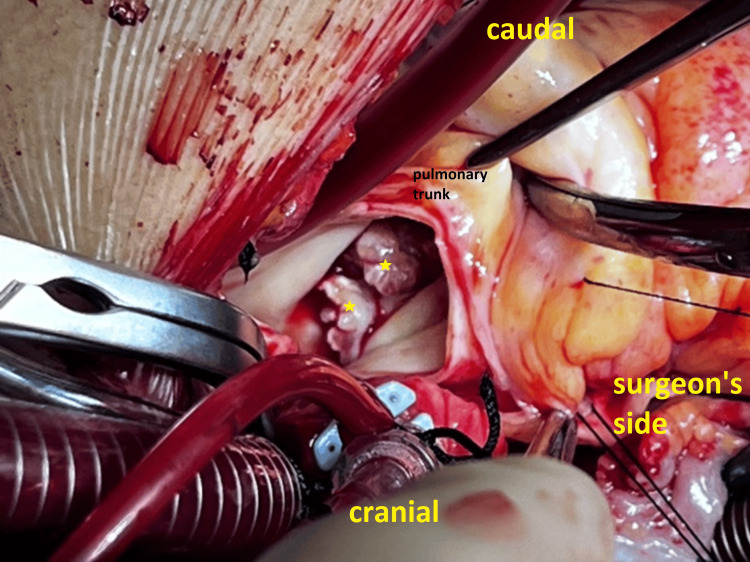
Image showing large pulmonary valve vegetations (yellow stars)

On the right side, large pulmonary valve vegetations were detected, with the largest measuring 39×15 mm (Figure 4).

**Figure 5:**
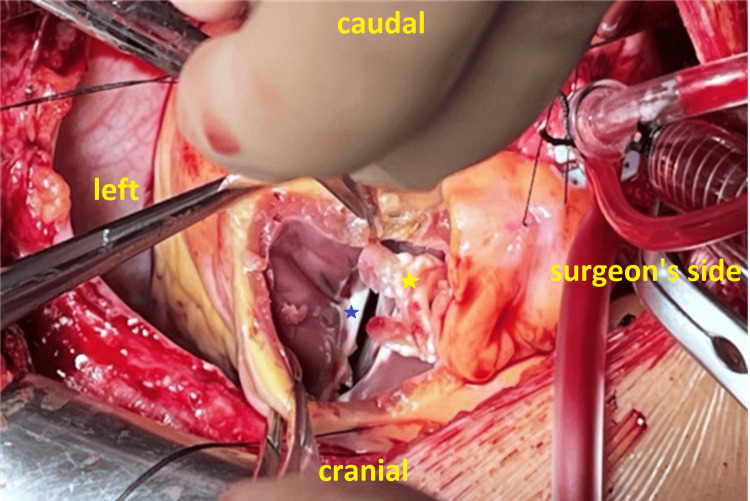
Image showing a fibromuscular narrowing in the right ventricular outflow tract indicating double outlet right ventricle (blue star) and vegetations in the right ventricular outflow tract (yellow star)

A fibromuscular narrowing was observed in the RVOT beneath the pulmonary valve with large vegetations, indicative of a DCRV (Figure 5).

The aortic valve was replaced with a mechanical valve (St. Jude Medical Regent, 23 mm, Saint Paul, Minnesota, United States), and the pulmonary artery was replaced with a stentless tissue valve (Medtronic Freestyle, 27 mm, Minneapolis, Minnesota, United States). The fibromuscular narrowing tissue was resected, resulting in a satisfactory RVOT diameter. The VSD was closed directly by incorporating it with the sutures of the aortic valve prosthesis (ETHICON Ethibond Excel 2/0, Raritan, New Jersey, United States).

Postoperatively, the patient was stable and maintained a normal sinus rhythm. Intraoperative specimen cultures were negative. Antibiotic therapy (vancomycin 30 mg/kg/day and ceftazidime 90 mg/kg/day) was extended for four weeks postoperatively, and the patient was discharged for regular follow-ups in our outpatient clinic.

## Discussion

IE remains a significant health issue, with a mortality rate of about 30% per year [10]. Based on the modified Duke criteria, diagnosis of IE relies on clinical examination, imaging, and blood cultures [9]. Generally, IE predominantly affects the left side of the heart. However, on the right side, the pulmonary valve is less commonly affected than the tricuspid valve [11,12]. Multivalvular IE is much rarer and is associated with a significant incidence of heart failure and complications requiring surgical intervention [8].

Congenital heart defects, especially VSDs, carry a higher risk of IE compared to the general population, as shown in the Swedish registry for congenital heart disease (SWEDCON) [5]. The VSD in our case is either congenital or secondary to endocarditis. The presence of features of a double outlet right ventricle (Figure 5), commonly associated with VSD, suggests that the VSD is longstanding and congenital in origin [13].

The mechanism of IE development in our case is unclear. However, blood turbulence across the VSD may generate negative pressure beneath the right coronary cusp, which predisposes to aortic valve regurgitation and leads to the formation of relatively large vegetations, often resulting in cusp perforation, as demonstrated in our case (Figure 3).

The development of IE on the right side, involving the pulmonary artery and RVOT, is probably related to the shunts created by the VSD and the DCRV. These conditions damage the endothelial lining of the pulmonary valve and RVOT, exposing them to bacterial colonization and vegetation formation.

Considering the patient's young age, we decided to implant a mechanical valve in the aortic position. Although tissue valves are prone to early degeneration in younger patients, mechanical valves provide long-term durability despite the need for lifelong anticoagulation therapy. Evaluating the benefits and risks, we proceeded with mechanical valve implantation. For the pulmonary position, due to the low-pressure gradient and the limited supply of homografts, we chose a tissue valve. In general, there is no significant difference in the durability between homografts and tissue valves in the pulmonary position [14]. 

## Conclusions

Despite its rarity, this case emphasizes the need to consider intracardiac shunts, particularly VSDs, as a potential cause of multivalvular endocarditis in patients presenting with suggestive symptoms.

In patients with known congenital heart defects, even if the defects are small, a high index of suspicion for endocarditis should be maintained when they present with suggestive symptoms.

Longstanding VSDs, frequently associated with features of a DCRV, may increase the risk of endocarditis, particularly in the pulmonary valve and subpulmonary region. Endocarditis prophylaxis in these patients is highly recommended.

Intraoperatively, a comprehensive transesophageal echocardiography (TEE) assessment and careful examination are crucial to avoid missing these shunts, which can result in increased postoperative morbidity and mortality.
